# A study of the car-to-train assignment problem for rail express cargos in the scheduled and unscheduled train services network

**DOI:** 10.1371/journal.pone.0204598

**Published:** 2018-10-10

**Authors:** Boliang Lin, Jingsong Duan, Jiaxi Wang, Min Sun, Wengao Peng, Chang Liu, Jie Xiao, Siqi Liu, Jianping Wu

**Affiliations:** 1 School of Traffic and Transportation, Beijing Jiaotong University, Beijing, China; 2 Department of Transportation, China Railway Guangzhou Group Co., Ltd., Guangzhou, Guangdong, China; University of Hong Kong, HONG KONG

## Abstract

A freight train service network generally involves two categories of trains: unscheduled trains, whose operating frequencies fluctuate with the freight demand, and scheduled trains, which are operated based on regular timetables similar to passenger trains. The timetables for scheduled trains are released to the public once determined, and they are not influenced by the freight demand. Typically, the total capacity of scheduled trains can satisfy the predicted average demand of express cargos. However, in practice, the demand always changes. Therefore, a method to assign the shipments to scheduled and unscheduled train services has become an important issue faced in railway transportation. This paper focuses on the coordinated optimization of rail express cargo assignment in a hybrid train services network. On the premise of fully utilizing the capacity of scheduled train services, we propose a car-to-train assignment model to reasonably assign rail express cargos to scheduled and unscheduled trains. The objective aims to maximize the profit of transporting the rail express cargos. The constraints include the capacity restriction on the service arcs, flow balance constraints, transportation due date constraints and logical relationship constraints among the decision variables. Furthermore, we discuss a linearization technique to convert the nonlinear transportation due date constraint into a linear constraint, making it possible to solve by a standard optimization solver. Finally, an illustrative case study based on the Beijing-Guangzhou Railway Line is carried out to demonstrate the effectiveness and efficiency of the proposed solution approach.

## Introduction

In recent years, the competition between railway and highway transportation has become increasingly intense, especially for the long-distance transportation of high-value freight. The governments expect that more freight flow transported by highways should be diverted to the railways in order to reduce carbon emissions. For example, in Europe, 30% of road freight over 300 km is expected to shift to railways or waterways by 2030. The Ministry of Transport of China also recommends that the railways and waterways should support more freight transportation loads [1]. However, road freight transport is convenient and efficient currently, and many shippers tend to choose the road transportation mode. Therefore, as one of the largest and busiest railway systems, the China Railway has focused on the development of scheduled train services in past years in an effort to obtain a higher market share in high-value freight transportation. For example, on the official website of the China Railway, we can find 156 scheduled train services, including trains that are bound for West Asia and Europe. The scheduled trains in China can be divided into the following three levels according to the operating speed: 160 km/h, 120 km/h and 80 km/h (the speed of an ordinary freight train is usually less than 50 km/h). The operating speed of scheduled trains is fast, which is beneficial to guarantee the delivery period of the shipments. Nevertheless, the high cost of new express train may exceed the income of the attracted cargos and may be not cost-effective. In addition, according to the current practice, some train schedule plan is usually made on the basis of historical data and the experience of the staff. However, some of the train services attract fewer freight flows than expected. In practice, an unreasonable plan can be improved only when a failure has occurred. Based on our previous estimation, the transportation demand of high-value freight is very large and is far beyond the supply of the 156 train services. Therefore, in this situation, it is necessary to improve the railway freight transport service to attract more freight flow from the road.

Currently, the trial and error based scheduled train service plan provided adopted by the railway company is myopic and is far from optimal. Therefore, it is an important task to evaluate whether the train service plan is reasonable enough as well as globally optimize the car-to-train plan using state-of-the-art methods. These important tasks were traditionally done manually, but there is an increasing move toward automated software based on mathematical models and algorithms. If mathematical models and algorithms can be conducted before the application of the train service plan, the decision failure can be reduced significantly. Therefore, optimizing the car-to-train assignment for the rail express cargo is an important problem that needs to be addressed both theoretically and practically.

## Related work

Generally, the core of the car-to-train assignment problem involves the two consolidation processes: the car-to-block assignment and the block-to-train assignment.

The objective of the blocking problem is to determine which blocks should be built at each yard and the assignment of cars to these blocks. The objective is to minimize the total cost. One of the first models was proposed by Bodin et al. [2], who developed a nonlinear model to determine a classification plan for all the classification yards, which can be viewed as a multicommodity flow problem with capacity constraints in terms of the maximum number of blocks and the maximum car volume that can be handled. Newton et al. [3] modeled the railway blocking problem as a network design problem with the objective of minimizing the total mileage, handling and delay costs. They developed a column generation, branch-and-bound algorithm to solve the model. In recent studies, the blocking problem is regarded as a very large-scale, multicommodity, service network design problem with billions of decision variables. Different heuristic optimization algorithms have been proposed to solve the problem. Barnhart [4] considered the flow constraints on the nodes and arcs and proposed a Lagrangian relaxation heuristic algorithm to solve the problem. The model was tested on a major railroad, and the validity of the model and algorithm were verified. Yaghini [5] presented an algorithm based on ant colony optimization to solve the blocking problem and compared the results with the solution generated with CPLEX software. The model was tested in Iran Railways, which reduced the operational cost considerably and saved time in shipping the freight. Gorman [6] addressed the joint train-scheduling and demand-flow problem for a major US freight railroad. A tabu-enhanced genetic search was used to find acceptable solutions, which consistently achieves better approximations to determine the optimum solution and maintains its performance as the problem size grows. Yaghini et al. [7] proposed a hybrid algorithm that integrated the Simplex method and simulated annealing to solve this problem. Ahuja et al. [8] developed the very large-scale neighborhood (VLSN) search algorithm to solve the blocking problem that can find the nearly optimal solution in one to two hours of computing time on a standard workstation computer. As the problem of air pollution is becoming increasingly severe compared to that of highway transport, the railway is a better choice with low energy consumption and carbon emissions. Lin et al. [1] and Liu et al. [9] considered the factor of carbon emissions in the railway network design problem and flow assignment model on railway and highway networks.

The block-to-train problem involves determining which train services need to be supplied at what frequency and assigning blocks to train services. Thomet [10] developed a cancellation procedure that gradually replaces direct trains with a series of intermediate train connections in order to minimize operation and delay costs. Kwona et al. [11] used a time-space network technique to improve a given blocking plan and the block-to-train assignment. The problem was formulated as a linear multicommodity flow problem, and the column generation technique was used as a solution approach. Jha et al. [12] provided two formulations for the block-to-train problem, including both an arc-based and a path-based time-space network; the path-based model was generally smaller than the arc-based model, and it can better handle practical constraints. The heuristic algorithm integrating a Lagrangian relaxation-based method and a greedy construction method was proposed to solve the path-based model, which can obtain an optimal solution within a few minutes of computational time. Xiao and Lin et al. [13] presented a comprehensive optimization model for the Train Formation Plan (TFP) network problem using both single-block trains and two-block trains. Compared to the single-block train service plan, they aimed to maximize the car-hour consumption savings at the yards. Based on the study of reference [13], Xiao et al. [14] defined an integer programming model to solve the block-to-train assignment problem aiming to maximize the total cost savings of the whole railroad network. The freight shipment demand for the railroad is increasing significantly and will cause severe congestion and inefficiency. Taesung and Ouyang [15] proposed a customized network assignment model that could capture traffic delays, and they adopted a convex combinations algorithm to determine the shipment routing equilibrium. Borndörfer et al. [16] also established a model aimed to minimize the sum of all expected delays and all running times, and they considered passenger trains.

Since the above two subproblems are interrelated, some researchers consider these two issues as an integrated problem and establish a car-to-train model directly to solve the train connection service problem. Keaton [17, 18] formulated the combined problem as a 0–1 mixed integer programming model that minimizes the sum of the train costs, car time costs, and classification yard costs, while not exceeding the limits on train size and yard volume. A heuristic approach based on Lagrangian relaxation was presented to solve the problem. Crainic et al. [19] formulated a general optimization model that takes into account the problem of routing traffic and scheduling train services. A heuristic algorithm was developed to solve this mixed-integer multicommodity problem. Haghani [20, 21] established a comprehensive model considering train routing and makeup and empty car routing in a space-time network. A heuristic decomposition technique was developed, which decomposes the problem into smaller subproblems based on the type of decision variables. Lin et al. [22] combined the blocking problem and the train makeup problem to build a bi-level linear integer model to solve the train service network problem of the China railway system and the simulated annealing algorithm is applied to real-life instances. Zhu et al. [23] addressed the scheduled service network design problem by proposing a model that combines service selection and scheduling, car classification and blocking, train makeup, and the routing of time-dependent customer shipments based on a cyclic three-layer space-time network representation of the associated operations and decisions and their relations and time dimensions. A methodology integrating slope scaling, a dynamic block generation mechanism, long-term memory-based perturbation strategies, and an ellipsoidal search was proposed, which is efficient and robust and yields high-quality solutions. Fügenschuh et al. [24] presented an integer programming formulation for the freight car-to-train routing problem that arises at Deutsche Bahn. The model aims at finding the car routes with the most economical cost, which considers the train and car travel kilometers and the number of used sorting tracks. Based on the high-speed railway network, Su et al. [25] put forward a multiobjective optimization model including the average travel time of trains, energy consumption and user satisfaction. An improved GA was employed to solve the train routing problem, and an improved GA based on train control was applied to a large-scale network. Moreover, Zhu [26, 27] proposed an efficient algorithm based on the Kuhn-Munkres (K-M) algorithm to solve role assignment problem, which is similar to the car-to-train assignment problem to some extent.

In this paper, we study the car-to-train assignment problem of rail express cargos on the mixed scheduled and unscheduled train services network and conduct an illustrative case study based on the Beijing-Guangzhou Railway Line. The contributions of this work to the existing research literature can be summarized as follows:

1. The operating frequencies of the unscheduled trains fluctuate with the freight demand, while the scheduled trains are operated based on regular timetables similar to the passenger trains; this paper coordinately considers the train service network that involves both the unscheduled trains and the scheduled trains.
2. Based on the mixed scheduled and unscheduled train service network, a car-to-train assignment model is formulated to assign the rail express cargos. Taking into account the characteristics of high value-added goods, the transportation due date constraint is added to the model, which includes the transportation time on the arcs and the transfer time between the arcs.
3. Due to the limited capacity of the scheduled train arcs and the relatively sufficient capacity of the unscheduled train arcs, when considering the capacity constraint of the arcs, we mainly aim at making full use of the scheduled trains’ capacities.

The remainder of this paper is organized as follows. Section 3 describes the car-to-train assignment problem of rail express cargos in detail. Section 4 provides a model formulation and its linearization techniques. A numerical example is conducted in Section 5. Finally, the conclusions of this paper are presented in Section 6.

## Problem descriptions

In this paper, a car is a rail vehicle or a wagon used as loading units for measuring the volume of shipments, while the train is consisted of multiple cars with the same or different destinations. A car-to-train assignment problem is to determine how to assign the shipments (cars) into the corresponding train services.

**Fig 1:**
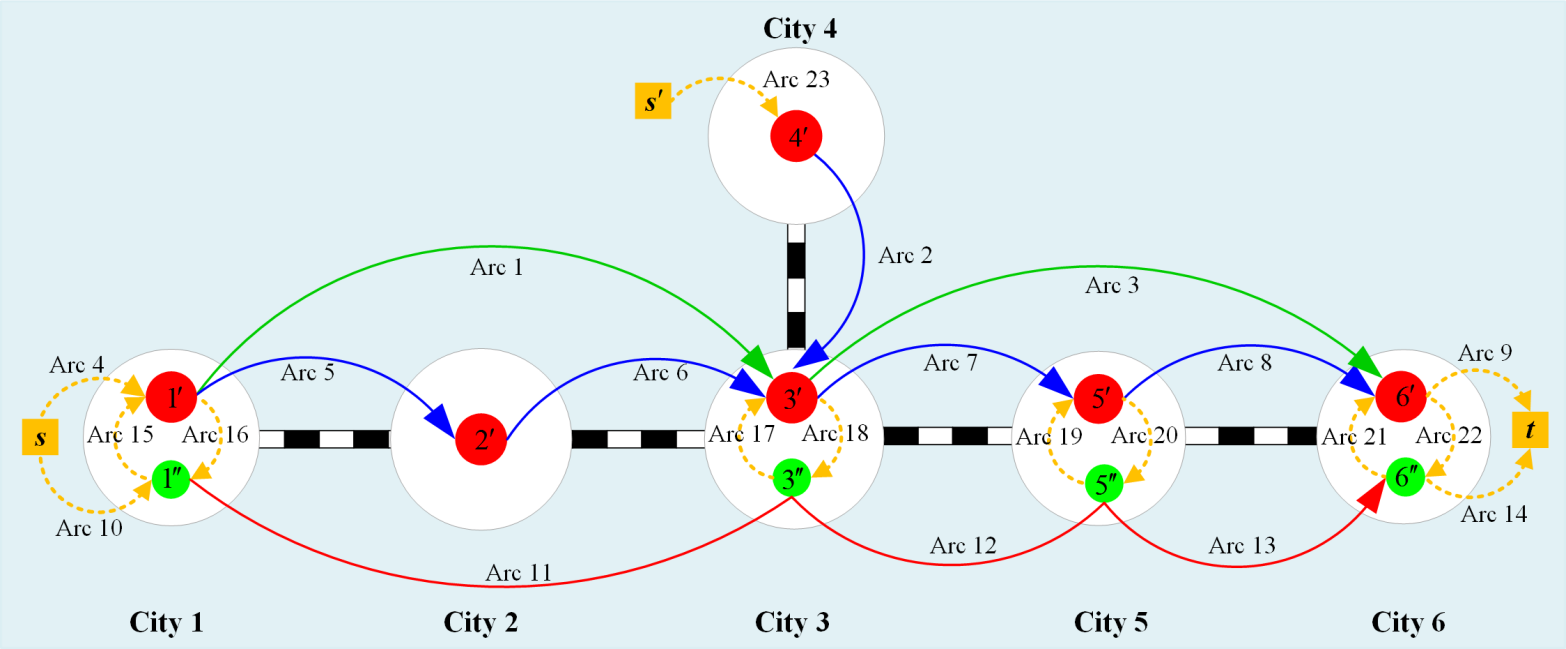
The railway service network.

A railway service network may consist of multiple types of train services, such as the local train services, the through train services and the express train services. Therefore, to transport a given shipment from its origin to its destination, we can use either a single type of train service or a combination of multiple train services. To better explain the overall transportation process, we provide more details using the example depicted in Fig 1.

In Fig 1, the red circles 1′~6′ represent railway classification yards, the green circles 1″, 3″, 5″ and 6″ are railway logistics centers, and the golden diamonds *s*, *s*′ and *t* denote big enterprises that have direct access (i.e., the enterprises’ special rail lines) to railways. The blue arcs Arc 2 and Arcs 5~8 represent shuttle train services (formed at one classification yard and broken up at the adjacent yard), and the green arcs Arc 1 and Arc 3 are through train services (i.e., long-distance ordinary train services that are formed at one classification yard, pass through one or more yards, and are finally broken up at a relatively far yard). Moreover, the yellow dotted arcs Arc 4, Arc 9, Arc 10, and Arcs 14~23 are local train services (or pickup and delivery train services between adjacent yards that are powered by shunting engines), and the red arcs Arc 11, Arc 12 and Arc 13 represent express train service arcs, which belong to the same express train service 1″→6″. Since the express train service 1″→6″ has car block swap operations at logistics centers 3″ and 5″, we decompose the train services into three connected service arcs.

Two shipments *s*→*t* and *s*′→*t* are considered, and their required transportation due dates are *T*_*st*_ and *T*_*s*′*t*_, respectively. The possible transportation strategies for the first shipment *s*→*t* were analyzed. One possible strategy is that the shipment is first transported to yard 1′ by the local train service Arc 4 from its origin *s* and is then transported to yard 3′ carried by the through train service Arc 1. After the classification operation, the shipment is grouped into the through train service Arc 3 and transported to the last yard 6′ on its itinerary. Finally, the shipment is sent to its destination *t* by the local train service Arc 9 to complete its entire itinerary. During the overall transportation process, the shipment is reclassified three times (at yards 1′, 3′ and 6′). The train service chain used by the train service is as follows:

1. *s*→Arc4→Arc1→Arc3→Arc9→*t*.

Other possible transportation strategies that can be adopted by the shipment include (but are not limited to) the following:

2 *s*→Arc4→Arc1→Arc7→Arc8→Arc9→*t*;
3 *s*→Arc4→Arc5→Arc6→Arc7→Arc8→Arc9→*t*; and
4 *s*→Arc10→Arc11→Arc12→Arc13→Arc14→*t*.

In Strategy (2), the shipment needs to be classified four times (at yards 1′, 3′, 5′ and 6′). Whereas in Strategy (3), the shipment needs to be classified five times (at yards 1′, 2′, 3′, 5′ and 6′). In contrast, only two classification operations (at yards 1′ and 6′) are needed in Strategy (4). However, delays occurred due to the car block swap operations at logistics centers 3″ and 5″ in this strategy, which may cause the transport time be more than the due date.

Similarly, for another shipment *s*′→*t*, the following transportation strategies can be adopted:

1. *s*′→Arc23→Arc2→Arc3→Arc9→*t*;
2. *s*′→Arc23→Arc2→Arc7→Arc8→Arc9→*t*; and
3. *s*′→Arc23→Arc2→Arc18→Arc12→Arc13→Arc14→*t*.

In Strategies (1) and (2), the shipment is reclassified three times and four times, respectively. In contrast, in Strategy (3), the shipment is reclassified three times and has one instance of a car block swap operation.

The train services are characterized by cost, operating speed, capacity and service frequency. For example, the express train service is usually of high cost, fast speed and low frequency, while the district train service has a lower speed at a lower cost and is always provided more frequently. As a result, some of the transportation strategies could violate the due date restrictions. Furthermore, some other strategies possibly result in overloads on the links through which the considered shipments and other shipments pass together. For instance, Strategy (1) of transporting shipment *s*→*t* and Strategy (1) of transporting shipment *s*′→*t* use the common through train service, i.e., Arc 3. If the volumes of these two shipments are relatively high, due to the limited capacity on Arc 3, its capacity constraint may be violated when the two abovementioned strategies are adopted simultaneously. Therefore, selecting the optimal transportation strategies for all the shipments while respecting the due date constraints and capacity constraints is typically a complicated combinatorial optimization problem.

## Mathematical model

This section aims to provide a mathematical description for the rail express cargo car-to-train assignment problem. The model aims at determining: (1) the portion of the freight volume of the high-value cargo that can be transported by the scheduled express trains (i.e. to evaluate whether the current train service plan is reasonable enough); (2) which train service arcs are used by each shipment (i.e. the train service chain of each shipment); (3) the workload (or capacity utilization rate) of the service arcs especially involved in the scheduled express trains. Our goal is to design an optimal car-to-train assignment plan that maximizes the net income of the railway company to deliver the rail express cargo. While the main constraints considered in our model include: transportation due date, train service arc capacity, flow balance at each station and logical relationship between decision variables.

To facilitate the model formulation, we make the following assumptions throughout this paper:

1. Each city has at least one railway station, and different stations (including marshalling stations and logistics centers) in a city can be connected by local train services.
2. Arcs between any two nodes are regarded as different arcs even if they belong to the same train services. For example, in Fig 1, though Arc 11 and Arc 12 belong to the same express train services, they are treated as two different arcs. Note that these two arcs are connected in Logistics Center 3″, where car block swap operations may occur.
3. Each shipment should not be split during the transportation process. This means that each shipment can only choose a single route (a train service chain). However, railway operators can decide whether to transport the whole volume of a shipment or just a portion of it.
4. The frequency of ordinary freight trains fluctuates with the traffic volume (once the volume reaches the predefined size of a train, an ordinary train will be dispatched. Thus, the number of trains dispatched per day fluctuates.). Because the traffic volume of each ordinary train arc is much larger than the express train arc, it is assumed that the traffic flows exceeding the capacity of an express train arc can definitely be transported by an ordinary train arc.

### Notations

The notations used in the mathematical model are described as follows:

Sets
  *V*: Set of all nodes, yards and logistics centers;
  *E*: Set of train service arcs;
  *E*^express^: Set of express train service arcs;
  *G*: Set of shipments, *g*∈*G*;
Parameters
  *s*_*m*_: Start node of an arc *m*, *s*_*m*_∈*V*, *m*∈*E*;
  *t*_*m*_: Tail node of an arc *m*, *t*_*m*_∈*V*, *m*∈*E*;
  *C*_*m*_: Capacity of an arc *m*;
  *L*_*m*_: Length of an arc *m*;
  *τ*_*m*_: Transportation time on an arc *m*;
  *τ*_*mn*_: Transfer time from arc *m* to arc *n* for a car;
  *N*_*g*_: Volume of the *g*th shipment that originates at *i* and is destined for *j*, *i*∈*V*_*supply*_, *j*∈*V*_*demand*_;
  *o*_*g*_: The origin of the *g*th shipment;
  *d*_*g*_: The destination of the *g*th shipment;
  *R*_*g*_: Per car cost of transporting the *g*th shipment paid by the shippers;
  *T*_*g*_: Transportation due date of the *g*th shipment;
  *λ*: The cost of the express train service per car kilometer;
  *M*: A sufficiently large number.
(3) Decision variables
  *ξ*_*g*_: Continuous variables. Proportion of the volume of the *g*th shipment that is able to be transported;
  $x_{g}^{m}$: Binary variables. $x_{g}^{m}=1$ if the *g*th shipment uses the *m*th service arc; otherwise, $x_{g}^{m}=0$.

### Model formulation

According to the notations above, the car-to-train assignment problem for the rail express cargo can be written as follows:

Objective function:

$$\max\sum_{g\in G}R_{g}N_{g}\xi_{g}-\lambda \sum_{m\in E^{\mathrm{express}}}C_{m}L_{m}$$ (1)

Subject to

$$\sum_{m\in E}x_{g}^{m}\tau_{m}+\sum_{m\in E}\sum_{n:s_{n}=t_{m}}\tau_{mn}x_{g}^{m}x_{g}^{n}\leq T_{g}\;\forall g\in G$$ (2)

$$\sum_{g\in G}N_{g}\xi_{g}x_{g}^{m}\leq C_{m}\;\forall m\in E$$ (3)

$$M\xi_{g}\geq x_{g}^{m}\;\forall g\in G,m\in E$$ (4)

$$\xi_{g}\sum_{m:s_{m}=o_{g}}x_{g}^{m}=\xi_{g}\;\forall g\in G,m\in E$$ (5)

$$\xi_{g}\sum_{m:t_{m}=d_{g}}x_{g}^{m}=\xi_{g}\;\forall g\in G,m\in E$$ (6)

$$\sum_{m:t_{m}=k}x_{g}^{m}=\sum_{n:s_{n}=k}x_{g}^{n}\;\forall g\in G,k\in V,k\neq o_{g},k\neq d_{g},m,n\in E$$ (7)

$$x_{g}^{m}\in \{0,1\}\;\forall g\in G,m\in E$$ (8)

$$\xi_{g}\in [0,1]\;\forall g\in G$$ (9)

In the model above, Eq (1) is the objective function, which maximizes the net income of transporting the rail express cargo. The net income is equal to the total transportation fees paid by the shippers minus the total operating costs of organizing the express train services by the railway company.

The second term in the objective function is the total operation cost of all express trains provided by Railway Company. The fixed operation cost of express train *i* is $C_{i}^{\mathrm{fix}}$, the per mile traveling cost is $C_{i}^{\mathrm{TrainRun}}$ and the transport distance is $l_{i}$. These parameters form the following equation:

$$\lambda \sum_{m\in E^{\mathrm{express}}}C_{m}L_{m}=\sum_{i\in S^{\mathrm{express}}}\left(C_{i}^{\mathrm{fix}}+C_{i}^{\mathrm{TrainRun}}l_{i}\right)$$ (10)

In formula (10), $S_{i}^{\mathrm{express}}$ is the set of express trains.

Express trains are usually operated based on regular timetables similar to passenger trains, they are not influenced by the freight demand. Even if the freight demand is very small, which rarely happens in practice because the express train schedule is made based on historical data, the train also has to be operated based on timetable and the railway company has to pay the operation cost. Therefore, the total cost of express trains can be viewed as a constant. The share of express cargo in the railway freight volume is small and very little of them is transported by the ordinary trains. Therefore, although the ordinary trains are operated based on freight demand, the express cargo volume has very limited influence on the frequencies of ordinary trains. As a result, the cost can be viewed as independent with the express cargo.

Since the service network is given in advance, the latter costs are a constant, which means it does not affect the optimal solution. Therefore, in the computational experiments, we will remove the constant express train service organization costs from the objective function.

Constraint (2) ensures that the total transportation time of a shipment should not be longer than its predefined due date. In this study, the total transportation time consists of the transportation time on the arcs and the transfer time between the arcs. Constraint (3) is the capacity restriction on the service arcs. Constraint (4) guarantees the logical relationship between the two groups of decision variables. Constraint (5)~(7) are the well-known flow balance constraints. Specifically, Constraint (5) is a flow balance constraint at the departure nodes, Constraint (6) is a flow balance constraint at the arrival nodes, and Constraint (7) is a flow balance constraint at the intermediate (passing) nodes. Finally, decision variable domains are specified by Constraints (8) and (9).

## Linearization

There exist several nonlinear constraints in the original model, including constraints (2), (3), (5) and (6). Clearly, due date constraint (2) is a nonlinear constraint because it involves the production of two decision variables. In general, a linear model is easier to solve than a nonlinear model. We are hence motivated to linearize original constraint (2) to a linear constraint. Therefore, we need to first introduce an auxiliary binary decision variable $y_{g}^{mn}$, and it is defined as follows:

$$y_{g}^{mn}=x_{g}^{m}\cdot x_{g}^{n}\;\forall g\in G,m\in E,n\in E,t_{m}=s_{n}$$ (11)

Hence, constraint (2) can be converted to:

$$\sum_{m\in E}x_{g}^{m}t_{m}+\sum_{m\in E}\sum_{n:s_{n}=t_{m}}\tau_{mn}y_{g}^{mn}\leq T_{g}\;\forall g\in G$$ (12)

In addition, we add the following constraints into the original model:

$$x_{g}^{m}+x_{g}^{n}-1\leq y_{g}^{mn}\leq \frac{1}{2}\cdot \left(x_{g}^{m}+x_{g}^{n}\right)\;\forall g\in G,m\in E,n\in E,t_{m}=s_{n}$$ (13)

$$y_{g}^{mn}\in \{0,1\}\;\forall g\in G,m\in E,n\in E,t_{m}=s_{n}$$ (14)

By replacing $x_{g}^{m}\cdot x_{g}^{n}$ with $y_{g}^{mn}$ in constraint (2) and adding constraints (13) and (14) to the original model, we transform constraint (2) to a linear constraint.

Then, we need to linearize constraints (3), (5) and (6). We introduce a continuous binary decision variable ${\bar{x}}_{g}^{m}$, and it is defined as follows:

$${\bar{x}}_{g}^{m}=\xi_{g}\cdot x_{g}^{m}\;\forall g\in G,m\in E$$ (15)

Hence, constraints (3), (5) and (6) can be converted to constraints (16), (17) and (18) respectively.

$$\sum_{g\in G}N_{g}{\bar{x}}_{g}^{m}\leq C_{m}\;\forall m\in E$$ (16)

$$\sum_{m:s_{m}=o_{g}}{\bar{x}}_{g}^{m}=\xi_{g}\;\forall g\in G,m\in E$$ (17)

$$\sum_{m:t_{m}=d_{g}}{\bar{x}}_{g}^{m}=\xi_{g}\;\forall g\in G,m\in E$$ (18)

In addition, we add the following constraints (19), (20) and (21) into the original model:

$$\xi_{g}-\left(1-x_{g}^{m}\right)M\leq {\bar{x}}_{g}^{m}\leq \xi_{g}+\left(1-x_{g}^{m}\right)M\;\forall g\in G,m\in E$$ (19)

$$-x_{g}^{m}M\leq {\bar{x}}_{g}^{m}\leq x_{g}^{m}M\;\forall g\in G,m\in E$$ (20)

$${\bar{x}}_{g}^{}\in [0,1]\;\forall g\in G$$ (21)

By replacing $\xi_{g}\cdot x_{g}^{m}$ with ${\bar{x}}_{g}^{m}$ in constraint (3), (5) and (6) and adding constraints (19), (20) and (21) to the original model, we transform constraint (3), (5) and (6) to linear constraints. Eventually, the original model is converted to a simpler mixed integer linear programming (MILP) model.

The car-to-train assignment problem can be viewed a generalization of the well-known Multicommodity Flow Problem (MFP). The MFP has already been proved to be NP-complete in existing literature. Therefore, the studied problem also belongs to the NP-complete class. Note that the studied problem is more complicated and computationally more challenging due to the additional transportation due date constraints. The solution method for our MILP model are branch-and-cut, and the commercially available solver Gurobi is employed in our computational study of this paper using its built-in branch-and-cut algorithm framework.

## Numerical results

**Fig 2:**
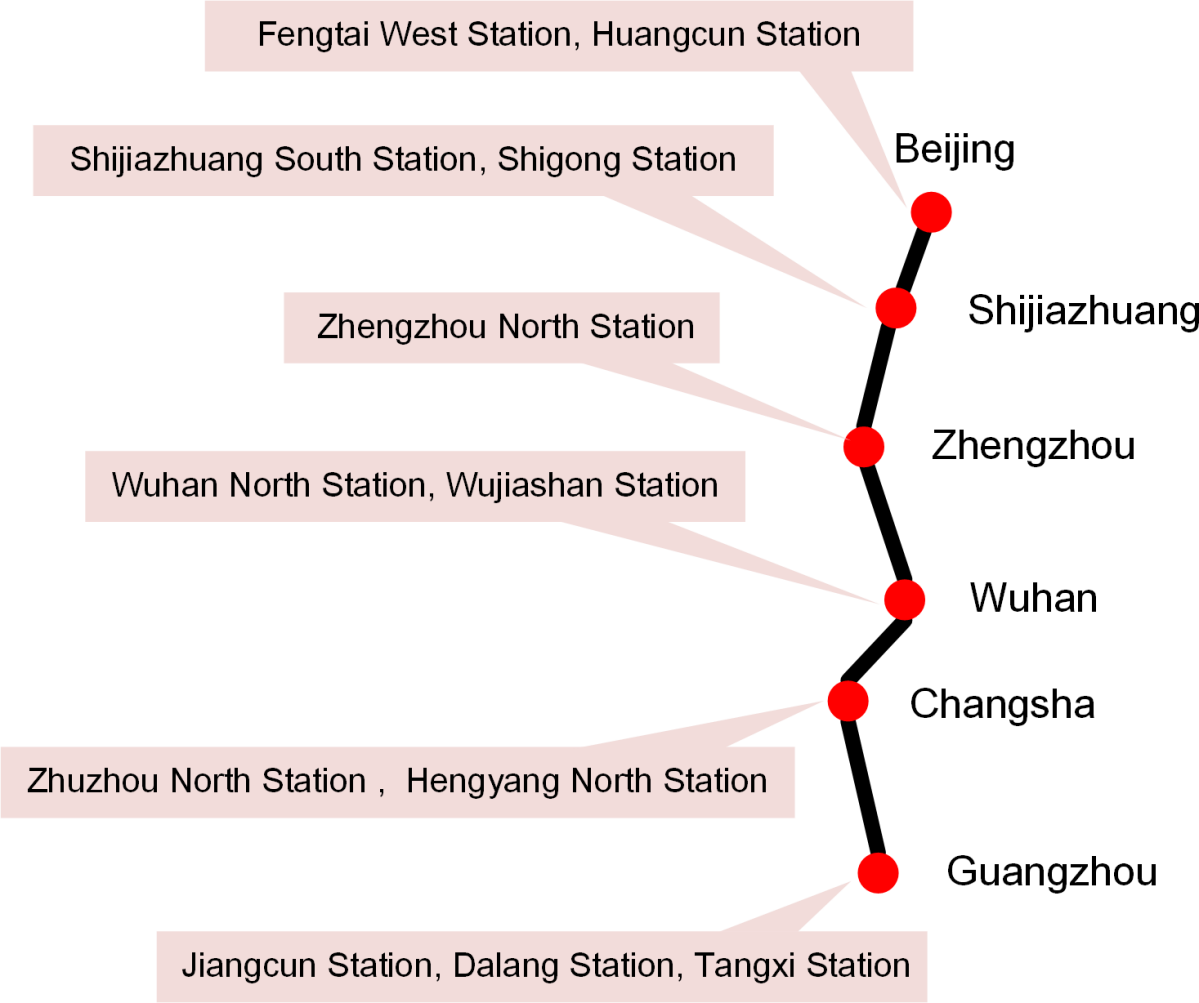
Beijing-Guangzhou railway line.

In this section, we perform computational analysis on a numerical example in the context of the railway corridor called “Beijing-Guangzhou Railway Line”, one of the most important north-south rail lines in China. The route, from Beijing to Guangzhou, has a total length of 2,324 kilometers. It connects 6 provincial capitals or municipalities and many large- and medium-sized cities, and it is one of the busiest major railways in China (see Fig 2). The goods transported to the south on the Beijing-Guangzhou railway mainly include coal, steel, petroleum, timber and export materials. Relatively speaking, the proportion of high value-added goods to the north is high. Therefore, this paper uses the northward direction of the Beijing-Guangzhou railway as an example for analysis.

This route passes through many important marshalling stations, including Fengtai West Station (Y007), Shijiazhuang South Station (Y006), Zhengzhou North Station (Y005), Wuhan North Station (Y004), Zhuzhou North Station (Y003), Hengyang North Station (Y002) and Jiangcun Station (Y001). We also consider some logistics centers related to this corridor, such as Dalang Station (B001) and Tangxi Station (B002) in Guangzhou, Wujiashan Station (B003) in Wuhan, Shigong Station (B004) at Shijiazhuang Hub, and Huangcun Station (B005) at Beijing Hub. In addition, the Nancang Station (B006) at Tianjin Hub is both an important marshalling station and a freight station. Although it does not belong to the Beijing-Guangzhou railway, due to its close relationship with Fengtai West Station, we also put it into the case system.

**Fig 3:**
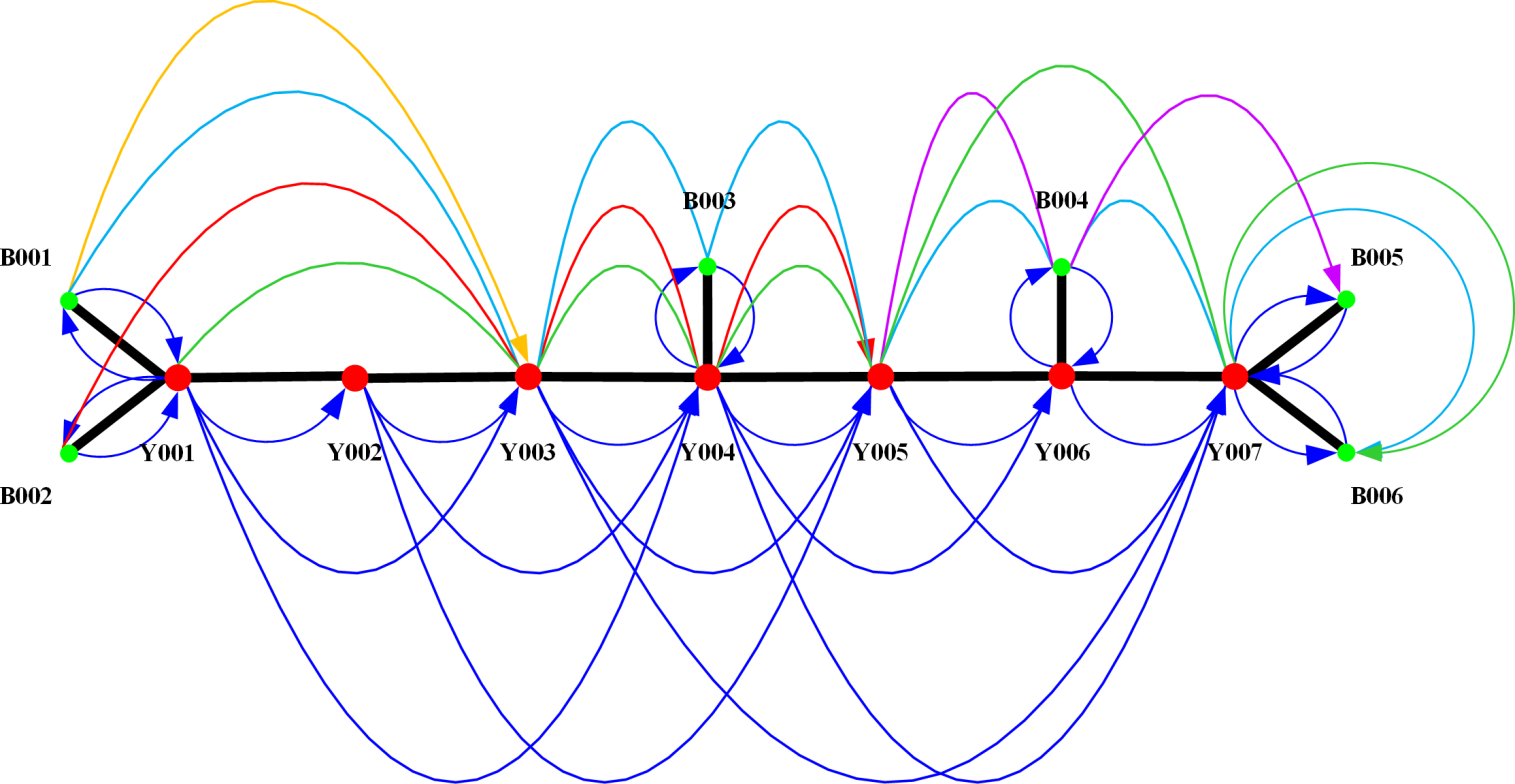
Service network for testing the model.

According to the data provided by the Railway Customer Service Center of China (12306 website), there are five express service trains (shown in Fig 3 as yellow, cyan, red, green and purple) related to the above marshalling stations and logistics centers. The curves of the same color belong to the same train, the point indicated by the arrow arc represents the destination of the train, and the point indicated by the curve without an arrow indicates the middle station of the train. For example, the red curve indicates the express train originating from B002, passing through Y003 and Y004 and terminating at Y005.

In addition, there are local trains running between the marshalling station and the logistics center and ordinary trains running from one marshalling station to another marshalling station (shown as blue arcs in Fig 3).

### Parameters of the model and information on the shipments

The freight demand, cost, and transportation due date for each station are shown in Table 1 for a total of 58 flows, with the flow ID from FLOW_01 to FLOW_58 corresponding to *g* = 1 to 58. The freight cost *R*_*g*_ is based on the Chinese Railway Tariff (see the data published on the website of 12306) and the transportation distance of shipment *g*. The average car volume in a day and freight transportation due date are analyzed and speculated based on historical data. Column *o*_*g*_ in Table 1 shows the origin station of the flow and column *d*_*g*_ indicates the destination station of the flow. The unit of car volume *N*_*g*_ is car, the unit of freight transportation due date *T*_*g*_ is hour, and the unit of freight cost *R*_*g*_ is CNY / car.

**Table 1:** Information on the shipments.

*g*	*o*_*g*_	*d*_*g*_	*N*_*g*_	*T*_*g*_	*R*_*g*_	*g*	*o*_*g*_	*d*_*g*_	*N*_*g*_	*T*_*g*_	*R*_*g*_
**FLOW_01**	B001	Y003	4.72	20	5598	**FLOW_30**	Y001	B006	5.28	72	19892
**FLOW_02**	B001	B005	14.64	72	19770	**FLOW_31**	Y001	B005	4.48	72	19808
**FLOW_03**	B001	Y004	4.48	26	9201	**FLOW_32**	Y002	Y004	3.76	24	5211
**FLOW_04**	B001	B003	29.44	36	9567	**FLOW_33**	Y002	B003	4.56	24	5577
**FLOW_05**	B001	Y005	13.76	34	13146	**FLOW_34**	Y002	Y005	4.24	29	9157
**FLOW_06**	B001	Y006	7.76	60	16363	**FLOW_35**	Y002	Y006	3.76	48	12373
**FLOW_07**	B001	B004	9.04	60	16603	**FLOW_36**	Y002	B004	13.68	60	12614
**FLOW_08**	B001	Y007	2.36	72	28534	**FLOW_37**	Y002	Y007	7.76	45	14434
**FLOW_09**	B001	Y007	7.68	72	19929	**FLOW_38**	Y002	B006	5.2	72	16645
**FLOW_10**	B001	B006	17.52	72	19854	**FLOW_39**	Y002	B005	5.04	60	16561
**FLOW_11**	B001	B006	2.15	72	28428	**FLOW_40**	Y003	Y005	25.92	24	19892
**FLOW_12**	B002	Y003	11.28	24	5673	**FLOW_41**	Y003	Y006	19.6	43	19808
**FLOW_13**	B002	Y004	8.56	36	9276	**FLOW_42**	Y003	B004	10.4	46	5211
**FLOW_14**	B002	B003	5.36	48	9642	**FLOW_43**	Y003	Y007	7.28	60	5577
**FLOW_15**	B002	Y005	5.12	36	13222	**FLOW_44**	Y003	B006	4.96	65	9157
**FLOW_16**	B002	Y006	3.76	60	16439	**FLOW_45**	Y003	B005	3.68	56	12373
**FLOW_17**	B002	B004	3.84	60	16679	**FLOW_46**	Y004	Y006	26.96	24	12614
**FLOW_18**	B002	B005	7.28	72	19694	**FLOW_47**	Y004	B004	4.8	32	14434
**FLOW_19**	B002	Y007	4.16	72	19853	**FLOW_48**	Y004	Y007	5.76	26	16645
**FLOW_20**	B002	Y007	8.72	72	28427	**FLOW_49**	Y004	B006	18.4	48	16561
**FLOW_21**	B002	B006	3.64	72	28321	**FLOW_50**	Y004	B005	12.24	48	8119
**FLOW_22**	B002	B006	17.44	72	19779	**FLOW_51**	B003	Y006	7.6	36	11336
**FLOW_23**	Y001	Y003	37.68	24	5560	**FLOW_52**	B003	B004	6.64	24	11576
**FLOW_24**	Y001	Y004	25.84	36	9163	**FLOW_53**	B003	Y007	5.92	36	13396
**FLOW_25**	Y001	B003	19.36	65	9529	**FLOW_54**	B003	B006	5.92	48	13322
**FLOW_26**	Y001	Y005	18.88	48	13108	**FLOW_55**	B003	B005	4.72	36	13237
**FLOW_27**	Y001	Y006	14.72	58	16325	**FLOW_56**	Y005	Y007	4.72	36	7732
**FLOW_28**	Y001	B004	8.72	48	16565	**FLOW_57**	Y005	B006	4.48	36	7973
**FLOW_29**	Y001	Y007	5.52	72	19966	**FLOW_58**	Y005	B005	3.84	36	9793

We classify the train service arcs in Fig 3, where ARC_01~ARC_16 are the service arcs of the express trains corresponding to *m* = 1~16 and ARC_17~ARC_43 are the service arcs of the ordinary trains corresponding to *m* = 17~43. The parameters of all the arcs are shown in Table 2.

**Table 2:** Information on the train services.

*m*	*s*_*m*_	*t*_*m*_	*τ*_*m*_	*C*_*m*_	Train _No.	*m*	*s*_*m*_	*t*_*m*_	*τ*_*m*_	*C*_*m*_	Train _No.
**ARC_01**	B001	Y003	7.3	20	X001	**ARC_23**	Y001	Y004	27.4	200	H007
**ARC_02**	B001	Y003	7.3	13	X002	**ARC_24**	Y002	Y003	3.3	200	H008
**ARC_03**	Y003	B003	7.9	27	X002	**ARC_25**	Y002	Y004	14.6	200	H009
**ARC_04**	B003	Y005	9.2	24	X002	**ARC_26**	Y002	Y005	27.1	200	H010
**ARC_05**	Y005	B004	6.7	19	X002	**ARC_27**	Y003	Y004	11.3	200	H011
**ARC_06**	B004	B006	5.4	21	X002	**ARC_28**	Y003	Y005	23.8	200	H012
**ARC_07**	Y005	B004	8.0	15	X003	**ARC_29**	Y003	Y007	40.5	200	H013
**ARC_08**	B004	B005	4.0	13	X003	**ARC_30**	Y004	B003	1.2	200	H014
**ARC_09**	B002	Y003	7.6	7	X004	**ARC_31**	B003	Y004	1.2	200	H015
**ARC_10**	Y003	Y004	7.0	12	X004	**ARC_32**	Y004	Y005	12.5	200	H016
**ARC_11**	Y004	Y005	10.0	13	X004	**ARC_33**	Y004	Y006	22.5	200	H017
**ARC_12**	Y001	Y003	7.5	25	X005	**ARC_34**	Y004	Y007	29.1	200	H018
**ARC_13**	Y003	Y004	12	25	X005	**ARC_35**	Y005	Y006	10.2	200	H019
**ARC_14**	Y004	Y005	7.5	25	X005	**ARC_36**	Y005	Y007	16.9	200	H020
**ARC_15**	Y005	Y007	9.4	25	X005	**ARC_37**	Y006	B004	1.4	200	H021
**ARC_16**	Y007	B006	4.0	25	X005	**ARC_38**	B004	Y006	1.4	200	H022
**ARC_17**	B001	Y001	0.5	200	H001	**ARC_39**	Y006	Y007	6.8	200	H023
**ARC_18**	Y001	B001	0.5	200	H002	**ARC_40**	Y007	B005	0.5	200	H024
**ARC_19**	B002	Y001	0.5	200	H003	**ARC_41**	Y007	B006	3.0	200	H025
**ARC_20**	Y001	B002	0.5	200	H004	**ARC_42**	B005	Y007	0.5	200	H026
**ARC_21**	Y001	Y002	12.8	200	H005	**ARC_43**	B006	Y007	3.0	200	H027
**ARC_22**	Y001	Y003	16.1	200	H006						

The columns *s*_*m*_ and *t*_*m*_ in Table 2 represent the origin and destination of the train service arc *m*, respectively, column *τ*_*m*_ represents the running time on arc *m* of the train, and column *C*_*m*_ represents the arc's capacity. Because the operating frequency of ordinary freight trains in China's rail system fluctuates with freight demands, we set the *C*_*m*_ value of the ordinary trains as 200 cars per day, which is equivalent to no capacity constraint. The column “Train _No.” is the arc belonging to the train trip.

The transfer time of Wuhan North Station and Hengyang North Station from 2005 to 2014 is listed in Table 3 (at this moment, the data after 2015 is not available). Wuhan North Station and Hengyang North Station are important marshalling stations on the Beijing-Guangzhou Railway Line. According to Table 3, the average transfer time is between 7.55 h and 8.30 h. The detailed data of all marshalling stations in the China railway system is not publically available. Therefore, we set the average transfer time 8 hours. Note that although this value may slightly change over different years, it has no essential influence on the mathematical modeling framework.

**Table 3:** The average transfer time of Wuhan North Station and Hengyang North Station from 2005 to 2014.

	2005	2006	2007	2008	2009	2010	2011	2012	2013	2014	Ave.
Wuhan North (hr)	8.70	8.50	8.60	8.50	8.80	8.00	8.30	7.80	8.00	7.80	8.30
Hengyang North (hr)	5.90	5.50	6.80	8.00	7.40	6.90	6.70	9.30	10.00	9.00	7.55

In fact, the transfer time value depends on all the shipments rather than one shipment. It is used to estimate the delivery time of cargos at the strategical level. In actual transportation, the transfer time of specific cargo may be influenced by many factors and it is complex to be accurately predicted and calculated. The problem in this paper falls into the strategical planning class, rather than detailed operational-level design of train timetables (schedules). The accurate value of transfer time for every shipment will be optimized at the operational level.

For different arcs belonging to the same train, the transfer time can be obtained from the express train timetable published on the 12306 website. Table 4 shows the values of the time delay parameter *τ*_*mn*_ of the transfer between two arcs, which have the same train number based on the train schedules. The unit of parameter *τ*_*mn*_ in Table 4 is hour.

**Table 4:** Transfer time between arc *m* and arc *n*.

Arc_m	Arc_n	*τ*_*mn*_	Arc_m	Arc_n	*τ*_*mn*_	Arc_m	Arc_n	*τ*_*mn*_
**ARC_02**	**ARC_03**	3.4	**ARC_07**	**ARC_08**	4.3	**ARC_13**	**ARC_14**	1.5
**ARC_03**	**ARC_04**	2.0	**ARC_09**	**ARC_10**	2.8	**ARC_14**	**ARC_15**	5.0
**ARC_04**	**ARC_05**	5.6	**ARC_10**	**ARC_11**	2.5	**ARC_15**	**ARC_16**	4.0
**ARC_05**	**ARC_06**	2.9	**ARC_12**	**ARC_13**	3.0			

### Computational results and analysis

We solve the model of the car-to-train assignment problem by Gurobi 7.5.2 implemented by Python 2.7.14. The parameters of the computer are Intel Core i5-3337U CPU and 4 GB RAM. The above instance based on the Beijing-Guangzhou railway line can be solved to optimality after running for 1747s. A total of 565.03 cars were involved in the calculation of the 58 flows, and the results show that 457.21 cars could be delivered within the prescribed due date, accounting for 79.16% of the total transportation demand, which is equivalent to ${\bar{R}}_{g}=79.16\%$. The corresponding objective function is 6140871.34 CNY. The detailed car-to-train assignment plan is shown in Table 5.

**Table 5:** Results of the car-to-train assignment.

*g*	*T*_*g*_	$T_{g}^{\prime }$	*ΔT*	*ξ*_*g*_	Car-to-Train Assignment Plan
**FLOW_01**	20	—	—	0.00%	——
**FLOW_02**	72	64.3	7.7	88.80%	B001→ARC_02→ARC_29→ARC_40→B005
**FLOW_03**	26	—	—	0.00%	——
**FLOW_04**	36	—	—	0.00%	——
**FLOW_05**	34	—	—	0.00%	——
**FLOW_06**	60	—	—	0.00%	——
**FLOW_07**	60	55.1	4.9	3.65%	B001→ARC_01→ARC_28→ARC_07→B004
**FLOW_08**	72	71.3	0.7	100.00%	B001→ARC_17→ARC_21→ARC_25→ARC_11→ARC_15→Y007
**FLOW_09**	72	68.1	3.9	100.00%	B001→ARC_17→ARC_12→ARC_13→ARC_34→Y007
**FLOW_10**	72	66.8	5.2	100.00%	B001→ARC_01→ARC_29→ARC_41→B006
**FLOW_11**	72	67.8	4.2	100.00%	B001→ARC_01→ARC_29→ARC_16→B006
**FLOW_12**	24	—	—	0.00%	——
**FLOW_13**	36	35.9	0.1	100.00%	B002→ARC_19→ARC_23→Y004
**FLOW_14**	48	45.1	2.9	100.00%	B002→ARC_19→ARC_23→ARC_30→B003
**FLOW_15**	36	—	—	0.00%	——
**FLOW_16**	60	58.2	1.8	18.35%	B002→ARC_19→ARC_12→ARC_13→ARC_14→ARC_35→Y006
**FLOW_17**	60	59.5	0.5	71.61%	B002→ARC_19→ARC_12→ARC_10→ARC_11→ARC_07→B004
**FLOW_18**	72	—	—	0.00%	——
**FLOW_19**	72	71.3	0.7	100.00%	B002→ARC_19→ARC_21→ARC_25→ARC_11→ARC_15→Y007
**FLOW_20**	72	65.8	6.2	100.00%	B002→ARC_19→ARC_21→ARC_25→ARC_14→ARC_15→Y007
**FLOW_21**	72	66.5	5.5	100.00%	B002→ARC_19→ARC_12→ARC_10→ARC_11→ARC_05 →ARC_06→B006
**FLOW_22**	72	67.1	4.9	40.14%	B002→ARC_09→ARC_29→ARC_41→B006
**FLOW_23**	24	16.1	7.9	100.00%	Y001→ARC_22→Y004
**FLOW_24**	36	27.4	8.6	100.00%	Y001→ARC_23→Y004
**FLOW_25**	65	36.6	28.4	100.00%	Y001→ARC_23→ARC_30→B003
**FLOW_26**	48	47.9	0.1	100.00%	Y001→ARC_21→ARC_25→ARC_32→B003
**FLOW_27**	58	57.9	0.1	100.00%	Y001→ARC_23→ARC_33→Y006
**FLOW_28**	48	46.2	1.8	39.45%	Y001→ARC_12→ARC_13→ARC_14→ARC_05→B004
**FLOW_29**	72	64.5	7.5	100.00%	Y001→ARC_21→ARC_25→ARC_34→Y007
**FLOW_30**	72	54.5	17.5	100.00%	Y001→ARC_12→ARC_13→ARC_14→ARC_05→ARC_06→B006
**FLOW_31**	72	58.9	13.1	33.93%	Y001→ARC_12→ARC_03→ARC_04→ARC_07→ARC_08→B006
**FLOW_32**	24	14.6	9.4	100.00%	Y001→ARC_25→Y004
**FLOW_33**	24	15.8	8.2	100.00%	Y002→ARC_25→ARC_30→Y003
**FLOW_34**	29	27.1	1.9	100.00%	Y002→ARC_25→ARC_32→Y005
**FLOW_35**	48	37.1	10.9	100.00%	Y002→ARC_25→ARC_33→Y006
**FLOW_36**	60	46.5	13.5	100.00%	Y002→ARC_25→ARC_33→ARC_37→B004
**FLOW_37**	45	43.7	1.3	100.00%	Y002→ARC_25→ARC_34→Y007
**FLOW_38**	72	54.7	17.3	100.00%	Y002→ARC_25→ARC_34→ARC_41→ARC_41→B006
**FLOW_39**	60	52.2	7.8	100.00%	Y002→ARC_25→ARC_34→ARC_40→B005
**FLOW_40**	24	23.8	0.2	100.00%	Y003→ARC_28→Y005
**FLOW_41**	43	41.8	1.2	100.00%	Y003→ARC_27→ARC_33→Y006
**FLOW_42**	46	39.8	6.2	100.00%	Y003→ARC_07→ARC_28→B004
**FLOW_43**	60	48.7	11.3	100.00%	Y003→ARC_28→ARC_36→Y007
**FLOW_44**	65	59.7	5.3	100.00%	Y003→ARC_28→ARC_36→ARC_41→B006
**FLOW_45**	56	49.0	7.0	100.00%	Y003→ARC_29→ARC_40→B005
**FLOW_46**	24	22.5	1.5	100.00%	Y004→ARC_33→Y006
**FLOW_47**	32	31.9	0.1	100.00%	Y004→ARC_33→ARC_37→B004
**FLOW_48**	26	21.9	4.1	100.00%	Y004→ARC_14→ARC_15→Y007
**FLOW_49**	48	40.1	7.9	100.00%	Y004→ARC_34→ARC_41→B006
**FLOW_50**	48	37.6	10.4	100.00%	Y004→ARC_34→ARC_40→B005
**FLOW_51**	36	31.7	4.3	100.00%	B003→ARC_31→ARC_33→Y006
**FLOW_52**	24	21.5	2.5	100.00%	B003→ARC_04→ARC_05→B004
**FLOW_53**	36	34.1	1.9	100.00%	B003→ARC_04→ARC_36→Y007
**FLOW_54**	48	46.1	1.9	100.00%	B003→ARC_04→ARC_36→ARC_16→B006
**FLOW_55**	36	33.5	2.5	84.75%	B003→ARC_04→ARC_15→ARC_40→B005
**FLOW_56**	36	16.9	19.1	100.00%	Y005→ARC_36→Y007
**FLOW_57**	36	27.9	8.1	100.00%	Y005→ARC_36→ARC_41→B006
**FLOW_58**	36	25.4	10.6	100.00%	Y005→ARC_36→ARC_40→B005

The column “Time” in Table 5 indicates the actual transportation time for the shipments. $\Delta T=T_{g}-T_{g}^{\prime }$ indicates the gap between the due date and the actual transportation time. According to Table 5, among the 58 flows, there are 42 flows that fully satisfy the demand, 8 flows partially meet the demand, and the remaining 8 flows cannot meet the demand. The reason can be analyzed as follows. For those paths satisfying the transportation due date constraints, the capacity of the arcs on these paths may be insufficient; for the other paths, the transportation due date constraints may be violated if they are used.

The car volume and workload of each arc are shown in Table 6. In the table, *f*_*m*_ indicates the actual car volume through arc *m*; *β*_*m*_ = *f*_*m*_/*C*_*m*_ is the workload of arc *m*. The average load of 16 express train arcs in the system is 75.54%. Among them, the workloads of 8 arcs reached the capacity values, and the workloads of ARC_03, ARC_06, ARC_08 and ARC_16 are all less than 50%. For ordinary train arcs, because they also transport other bulk non-value-added car flows, their workloads are not discussed here.

**Table 6:** Workloads of the express train arcs.

*m*	*f*_*m*_	*C*_*m*_	*β*_*m*_	*m*	*f*_*m*_	*C*_*m*_	*β*_*m*_
**ARC_01**	20.00	20	100.00%	**ARC_09**	7.00	7	100.00%
**ARC_02**	13.00	13	100.00%	**ARC_10**	6.39	12	53.25%
**ARC_03**	1.52	27	5.63%	**ARC_11**	12.91	13	99.31%
**ARC_04**	24.00	24	100.00%	**ARC_12**	25.00	25	100.00%
**ARC_05**	19.00	19	100.00%	**ARC_13**	17.09	25	68.36%
**ARC_06**	8.92	21	42.48%	**ARC_14**	23.89	25	95.56%
**ARC_07**	15.00	15	100.00%	**ARC_15**	25.00	25	100.00%
**ARC_08**	1.52	13	11.69%	**ARC_16**	8.07	25	32.28%

**Fig 4:**
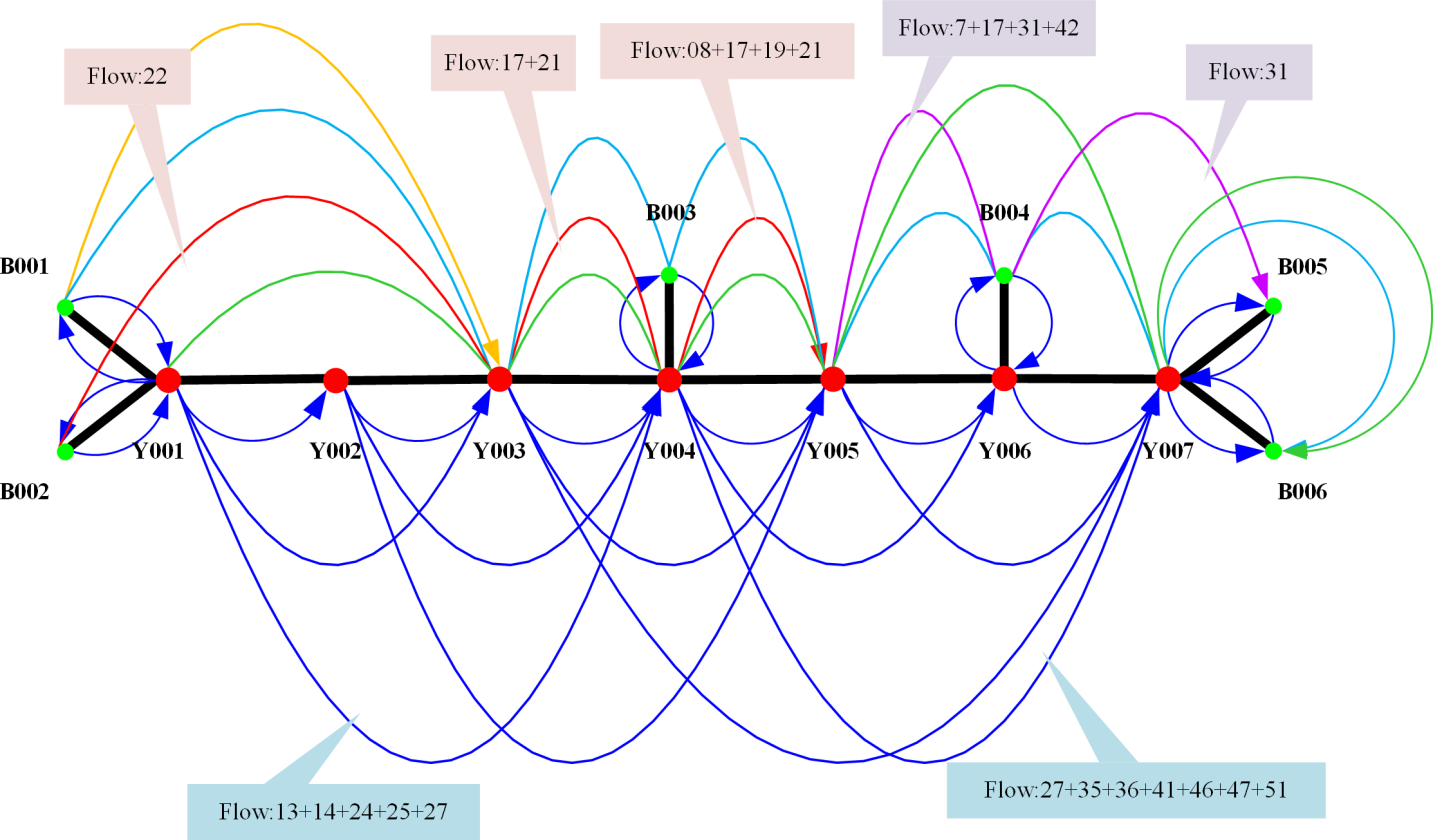
Assignment results of a part of the arcs.

The flow pattern of each arc (i.e., which shipments are carried by the train) is shown in Fig 4. In Fig 4, we select two express trains and two ordinary trains for illustration. The express train X003 (shown by red arcs in Fig 4) originates at station B002 and ends at station Y005. The flow it loads in the first segment of the trip (B002→Y003) is FLOW_22, the flows in the second segment are FLOW_17 and FLOW_21, and those in the third segment are FLOW_08, FLOW_17, FLOW_19 and FLOW_21. Similarly, the flows carried by express train X003 (shown by purple arcs in Fig 4) originate at station Y005 and end at station B005; the flows in the first segment of the trip (Y005→B004) are FLOW_7, FLOW_17, FLOW_31 and FLOW_42; the second segment is FLOW_31. The ordinary train (Y001→Y004) transports FLOW_13, FLOW_14, FLOW_24, FLOW_25 and FLOW_27. The ordinary train (Y003→Y007) transports FLOW_27, FLOW_35, FLOW_36, FLOW_41, FLOW_46, FLOW_47 and FLOW_51.

## Conclusions

In this paper, a car-to-train assignment model for rail express cargos is proposed. According to the characteristics of high value-added goods, the transportation due date constraint is added to the model, considering both the transportation time on the arcs and the transfer time between the arcs. In addition, the arc capacity constraint and flow balance constraint are considered. Moreover, linearization techniques are used to make the model linear so that it can be directly solved by a standard optimization solver. Finally, an illustrative case study is carried out based on the Beijing-Guangzhou Railway Line, which is one of the busiest railway lines in China. The example consists of 13 nodes and 43 arcs, and we consider the northward direction. The detailed car-to-train assignment plan is obtained based on the analysis of the optimal solution. The results indicate that the model has a certain significance in real-life application to the rail express cargos assignment problem.

Note that it is assumed that the train operation plan, mainly the scheduled train services network, is given, which is developed manually in practice without any optimization-based approach. In this case, the resulting car-to-train assignment plan can serve as a solid aid in improving the quality of train operation planning. In the long term, researchers can focus on the joint optimization of the train operation plan and the car-to-train assignment plan. In addition, the railway network and the cargo volume in China are huge. For such a large-scale network and data, the efficiency of the algorithm in this paper is far from enough for practical application, so the solution method of the model needs to be further improved.

## References

[1] Liu C, Lin BL, Wang JX, Xiao J, Liu SQ. Flow assignment model for quantitative analysis of diverting bulk freight from road to railway. Plos One. 2017;12(8):e0182179 10.1371/journal.pone.0182179 28771536 PMC5542531

[2] Bodin LD, Golden BL, Schuster AD. A model for the blocking of trains. Transportation Research Part B: Methodological. 1980;14(1–2):115–120. 10.1016/0191-2615(80)90037-5

[3] Newton HN, Barnhart C, Vance PH. Constructing railroad blocking plans to minimize handling costs. Transportation Science. 1998;32(4):330–345. 10.1287/trsc.32.4.330

[4] Barnhart C, Jin H, Vance PH. Railroad blocking a network design application. Operations Research. 2000;48(4):603–614. 10.1287/opre.48.4.603.12416

[5] Yaghini M, Foroughi A, Nadjar B. Solving railroad blocking problem using ant colony optimization algorithm. Applied Mathematical Modelling. 2011;35(12):5579–5591. 10.1016/j.apm.2011.05.018

[6] Gorman MF. An application of genetic and tabu searches to the freight railroad operating plan problem. Annals of Operations Research. 1998;78(0):51–69. http://hdl.handle.net/10.1023/A:1018906301828

[7] Yaghini M, Momeni M, Sarmadi M. Solving train formation problem using simulated annealing algorithm in a simplex framework. Journal of Advanced Transportation. 2014;48(5):402–416. 10.1002/atr.1183

[8] Ahuja RK, Jha KC, Liu J. Solving real-life railroad blocking problems. Interfaces. 2007;37(5): 404–419. 10.1287/inte.1070.0295

[9] Lin BL, Liu C, Wang HJ, Lin RX. Modeling the railway network design problem: A novel approach to considering carbon emissions reduction. Transportation Research Part D: Transport and Environment. 2017;56:95–109. 10.1016/j.trd.2017.07.008

[10] Thomet MA. A User-Oriented Freight Railroad Operating Policy. IEEE Transactions on Systems, Man, and Cybernetics. 1971;SMC-1(4):349–356. 10.1109/TSMC.1971.4308318

[11] Kwona OK, Martlandb CD, Sussmanb JM. Routing and scheduling temporal and heterogeneous freight car traffic on rail networks. Transportation Research Part E: Logistics and Transportation Review. 1998;34 (2):101–115. 10.1016/S1366-5545(97)00022-7

[12] Jha KC, Ahuja RK, Sahin G. New approaches for solving the block-to-train assignment problem. Networks. 2008;51(1):48–62. 10.1002/net.20195

[13] Xiao J, Lin BL. Comprehensive optimization of the one-block and two-block train formation plan. Journal of Rail Transport Planning & Management. 2016;6(3):218–236. 10.1016/j.jrtpm.2016.09.002

[14] Xiao J, Joern P, Lin BL, Wang JX. Solving the block-to-train assignment problem using the heuristic approach based on the genetic algorithm and tabu search. Transportation Research Part B: Methodological. 2018;108:148–171. 10.1016/j.trb.2017.12.014

[15] Hwang T, Ouyang Y. Assignment of Freight Shipment Demand in Congested Rail Networks. Transportation Research Record: Journal of the Transportation Research Board. 2014;2448(-1):37–44. 10.3141/2448-05

[16] Borndörfer R, Klug T, Schlechte T, Fügenschuh A, Schang T, Schülldorf H. The Freight Train Routing Problem for Congested Railway Networks with Mixed Traffic. Transportation Science. Published online in Articles in Advance 31 Mar 2016. 10.1287/trsc.2015.0656

[17] Keaton MH. Designing Optimal Railroad Operating Plans: Lagrangian Relaxation and Heuristic Approaches. Transportation Research Part B: Methodological. 1989;23(6):415–431. 10.1016/0191-2615(89)90042-8

[18] Keaton MH. Designing Railroad Operating Plans: A Dual Adjustment Method for Implementing Lagrangian Relaxation. Transportation Science. 1992;26(4):263–279. 10.1287/trsc.26.4.263

[19] Crainic T, Ferland JA, Rousseau JM. A Tactical Planning Model for Rail Freight Transportation. Transportation Science. 1984;18 (2):165–184. 10.1287/trsc.18.2.165

[20] Haghani AE. Rail freight transportation: A review of recent optimization models for train routing and empty car distribution. Journal of Advanced Transportation. 1987;21(2):147–172. 10.1002/atr.5670210205

[21] Haghani AE. Formulation and solution of a combined train routing and makeup, and empty car distribution model. Transportation Research Part B: Methodological. 1989;23(6):433–452. 10.1016/0191-2615(89)90043-X

[22] Lin BL, Wang ZM, Ji LJ, Tian YM, Zhou GQ. Optimizing the freight train connection service network of a large-scale rail system. Transportation Research Part B: Methodological. 2012;46(5):649–667. 10.1016/j.trb.2011.12.003

[23] Zhu E, Cranic TG, Gendreau M. Scheduled Service Network Design for Freight Rail Transportation. Operations Research. 2014;62(2):383–400. 10.1287/opre.2013.1254

[24] Fügenschuh A, Homfeld H, Schülldorf H. Single-Car Routing in Rail Freight Transport. Transportation Science. 2015;49(1):130–148. 10.1287/trsc.2013.0486

[25] Sun Y, Cao C, Wu C. Multi-objective optimization of train routing problem combined with train scheduling on a high-speed railway network. Transportation Research Part C: Emerging Technologies. 2014;44:1–20. 10.1016/j.trc.2014.02.023

[26] Zhu HB. Avoiding Conflicts by Group Role Assignment. IEEE Trans. on Systems, Man, and Cybernetics: Systems. 2016;46(4):535–547.doi: 10.1109/tsmc.2015.2438690

[27] Zhu HB, Zhou MC, Alkins R. Group Role Assignment via a Kuhn-Munkres Algorithm-based Solution. IEEE Trans. on Systems, Man, and Cybernetics, Part A: Systems and Humans. 2012;42(3):739–750.doi: 10.1109/tsmca.2011.2170414

